# Effect of Hepatic Impairment on the Pharmacokinetics of Baicalin in Rats: Critical Roles of Gut Microbiota and Hepatic Transporters

**DOI:** 10.3390/pharmaceutics17070851

**Published:** 2025-06-29

**Authors:** Ping Li, Yihua Tian, Hong Wang, Yuting Ji, Huiying Zeng, Shengman Zhang, Xiuli Gao, Xiaoyan Chen

**Affiliations:** 1 School of Pharmaceutical Sciences, Guizhou Medical University, Guiyang 561113, China; liping1@zidd.ac.cn (P.L.); 18815139838@163.com (Y.T.); 2 Zhongshan Institute for Drug Discovery, Zhongshan 528437, China; zenghuiying@zidd.ac.cn; 3 State Key Laboratory of Drug Research, Shanghai Institute of Materia Medica, Chinese Academy of Sciences, Shanghai 201203, China; s20-wanghong@simm.ac.cn (H.W.); zhangshengman@sjtu.edu.cn (S.Z.); 4 School of Pharmaceutical Sciences, Southern Medical University, Guangzhou 510515, China; jiyuting@zidd.ac.cn

**Keywords:** baicalin, hepatic impairment, pharmacokinetics, gut microbiota, hepatic transporters, enterohepatic circulation

## Abstract

**Background**: Baicalin (BG) has been used in the treatment of many diseases. However, the effect of hepatic insufficiency on its pharmacokinetics has not been reported, and there is a lack of clinical guidance for the use of BG in patients with hepatic impairment. **Methods**: Carbon tetrachloride (CCl_4_)-induced rat models were used to simulate hepatic failure patients to assess the effect of hepatic impairment on the pharmacokinetics and distribution of BG. In vitro metabolism and transporter studies were employed to elucidate the potential mechanisms. **Results**: After intragastric administration of 10 mg/kg of BG, the peak plasma concentration and exposure (AUC_0–t_) of BG decreased by 64.6% and 52.6%, respectively, in CCl_4_-induced rats. After intravenous administration, the AUC_0–t_ decreased by 73.6%, and unlike in the control group, the second absorption peak of BG was not obvious in the concentration–time curve of CCl_4_-induced rats. The cumulative excretion of BG in the feces increased, but that in the bile decreased. In vivo data indicated that the absorption and enterohepatic circulation of BG were affected. In vitro studies found that the hydrolysis of BG to the aglycone baicalein decreased significantly in the intestinal tissues and contents of the CCl_4_-induced rats. And BG was identified as a substrate for multiple efflux and uptake transporters, such as breast cancer resistance protein (BCRP) and multidrug resistance-associated proteins (MRPs), organic anion transporting polypeptides (OATP1B1, 1B3, 2B1), and organic anion transporters (OATs). The bile acids accumulated by liver injury inhibited the uptake of BG by OATPs, especially that by OATP2B1. **Conclusions**: Hepatic impairment reduced BG hydrolysis by intestinal microflora and inhibited its transporter-mediated biliary excretion, which synergistically led to the attenuation of the enterohepatic circulation of BG, which altered its pharmacokinetics.

## 1. Introduction

Baicalin (5,6-dihydroxy-flavone-7-*O*-glucuronide, BG) is the most effective bioactive component of the roots of *Scutellaria baicalensis*. It has been reported to exhibit various pharmacological functions, including antioxidant, anti-tumor, anti-inflammatory, antibacterial, antiviral (e.g., COVID-19 treatment), and hepatoprotection effects [1,2,3]. BG is the main active ingredient in a variety of proprietary Chinese medicines, such as Shuanghuanglian oral liquid, BG tablets, Huanglian Shangqing pills, and Yingqiaojiedu mixtures, which are widely utilized in clinical practice for the treatment of upper respiratory tract infections, acute tonsillitis, pharyngitis, and other conditions.

As a representative flavonoid, the in vivo process of BG has been extensively reported. Currently, accumulating evidence has demonstrated that BG is poorly hydrophilic and lipophilic [3], with metabolic enzymes and multiple transporters, such as breast cancer resistance protein (BCRP), multidrug resistance-associated proteins (MRPs), organic anion transporters (OATs), and organic anion transporting polypeptides (OATPs) [4,5,6,7,8] involved in its absorption and disposition. After oral administration, a small portion of BG was directly taken up into the enterocytes by OATPs (OATP2B1, 1B1, and 1B3) in the duodenum [5,6], while the majority was hydrolyzed to the aglycone baicalein (B) by intestinal microbiota-derived β-glucuronidase and subsequently absorbed [9,10,11]. B was then metabolized by enterocyte UDP-glucuronosyltransferases (UGTs), mainly by UGT1A9, to protype BG [12]. BG generated via metabolism was partially excreted back into the intestinal lumen by efflux transporters (BCRP and MRP2), forming an entero-intestinal cycle. The remaining BG was absorbed into the liver and subsequently entered the systemic circulation or was excreted into bile via BCRP and MRP2, thereby returning to the duodenum and forming enterohepatic circulation [4,13].

Chronic hepatic impairment, resulting from viral liver disease, non-alcoholic fatty liver disease, and alcoholic liver disease leads, to hepatocyte damage, necrosis, and morphological and structural destruction of the liver, which can cause dysfunctions in drug absorption and disposition [14,15]. Consequently, medication optimization may be necessary for these patients. In the case of verapamil, hepatic impairment reduces its metabolism and prolongs its elimination half-life. Thus, patients with severe hepatic insufficiency receive only about 30% of the dose given to those with normal hepatic function. Although BG has been used clinically for many years, it remains unclear whether liver dysfunction affects the absorption and disposition in vivo and whether dose adjustment is needed in such patients.

Carbon tetrachloride (CCl_4_) has been commonly used as an inducer of acute or chronic hepatic impairment in animal models (mice, rats, or rabbits), simulating the pathological process of liver fibrosis in patients with chronic hepatic impairment. In this study, we constructed a rat model of CCl_4_-induced hepatic impairment, combined with in vitro experiments, to evaluate the effect of hepatic impairment on the pharmacokinetics and distribution of BG, elucidating the effect mechanism.

## 2. Materials and Methods

### 2.1. Chemicals and Reagents

BG (99.5% purity) and its aglycone B (98.2% purity) were purchased from Aladdin Biochemical Technology Co., Ltd. (Shanghai, China). Verapamil (99.1% purity, internal standard), Tris-HCl, Hanks’ balanced salt solution (HBSS), the BCA protein assay kit, and phosphate buffer (PBS, pH7.4) were obtained from Meilun Biology Technology (Dalian, China). 6-hydroxy-flavone (purity 97.8%, internal standard) was purchased from Bailingwei Technology Co., Ltd. (Beijing, China). CCl_4_, vitamin C, and corn oil were supplied by Aladdin Biochemical Technology Co., Ltd. (Shanghai, China). Alanine aminotransferase (ALT), aspartate aminotransferase (AST), and alkaline phosphatase (AKP) assay kits of were purchased from Nanjing Jiancheng Bioengineering Institute (Nanjing, Jiangsu, China). Alamycin, uridine 5′-diphosphoglucuronic acid (UDPGA), rifampicin, and probenecid were purchased from Sigma-Aldrich (St. Louis, MO, USA). Dulbecco’s modified Eagle’s medium (DMEM), fetal bovine serum (FBS), trypsin, nonessential amino acids, streptomycin, and penicillin G were sourced from Invitrogen (Carlsbad, CA, USA). Bile acids were purchased from Yuanye Biotechnology Co., Ltd. (Shanghai, China). Purified water was obtained using a Millipore Milli-Q gradient water purification system (Molsheim, France). All other solvents and reagents were of chromatographic or analytic grade.

### 2.2. Construction of CCl_4_-Induced Hepatic Failure Rat Model

Male Sprague Dawley (SD) rats weighing 180 to 220 g were randomized into two groups. The CCl_4_-treated rats were administered an intraperitoneal injection of CCl_4_ (20% in corn oil) at a dose of 2.5 mL/kg every 2 days for 8 weeks. The control rats were treated intraperitoneally with the same volume of corn oil at the same time intervals. The successful model of CCl_4_-induced rats with liver injury was judged by measuring the concentration of ALT, AST, and AKP in serum and liver tissues stained with hematoxylin–eosin (HE).

### 2.3. Pharmacokinetic Experiments

Rats were fasted for 12 h with free access to water before the experiment. BG at 10 mg/kg and equimolar B (6.05 mg/kg) were administered orally (prepared in 0.5% carboxymethyl cellulose sodium) or intravenously (dissolved in 5% dimethyl sulfoxide, 5% ethyl alcohol, 40% PEG 300 and 50% sterile H_2_O) to CCl_4_-induced rats (*n* = 5) and control rats (*n* = 5). Blood was collected from the retro-orbital venous plexus before administration (0 h), at 5, 15, 30 min, and 1, 2, 3, 4, 5, 6, 8, 12, 24, 48, and 72 h post-dosing in tubes containing EDTA-2K. Plasma samples obtained by centrifugation at 11,000× *g* for 5 min at 4 °C were immediately mixed with 10% 2 M (mol/L) vitamin C (1/20, *v*/*v*) and then stored at −80 °C until further analysis.

### 2.4. Excretion Experiments

The two groups of rats (*n* = 5) were placed into individual metabolic cages, fasted for 12 h before administration and were allowed to drink freely. BG and B were administered orally or intravenously, as above. Feces and urine samples were collected before (0 h) and at 0–6, 6–10, 10–13, 13–24, 24–36, and 36–48 h after dosing. The weight of the fecal samples and the urine volume from each period were recorded. Then urine samples were immediately acidified to pH 2 with 8 M phosphoric acid and then stored at −80 °C. The fecal samples were homogenized and extracted in a five-fold volume (1:5, *w*/*v*) of acetonitrile-H_2_O (1:1, *v*/*v*, containing 10% 8 M phosphoric acid) four times before analysis.

To collect bile samples, both the CCl_4_-induced rats and control rats underwent common bile duct intubation surgery prior to dosing. Bile samples were collected with PE-50 polyethylene tubing before administration and at 0–2, 2–4, 4–8, 8–11, 11–24, 24–34, and 34–48 h after an oral administration of 10 mg/kg BG. The bile volume from each period was recorded, then immediately acidified to pH 2 with 8 M phosphoric acid and stored at −80 °C. In order to evaluate the effect of liver injury on BG enterohepatic circulation, plasma samples were also collected from these duct-cannulated rats, as described in Section 2.3.

### 2.5. Tissue Distribution Experiment

BG was orally administered to CCl_4_-induced rats and control rats at 10 mg/kg. At 0.5, 3, 8, and 24 h post-dosing; the rats (*n* = 3/group/time) were anesthetized with pentobarbital sodium and sacrificed to collect the duodenum, jejunum, ileum, colon, and the contents of each intestinal segment separately. The tissues were harvested, weighed, and rinsed with ice-cold saline and then frozen at −80 °C for further analysis. The intestinal tissues and contents were homogenized in a five-fold volume (1:5, *w*/*v*) of acetonitrile-H_2_O (1:1, *v*/*v*, containing 10% 8 M phosphoric acid) before analysis.

### 2.6. Intestinal Flora Incubation

The contents of the duodenum, jejunum, ileum, cecum, and colon from the CCl_4_-induced rats and control rats were collected in PE-50 polyethylene tubing using sterile liquid (0.9% saline, 20% glycerol, pH 7.2). The intestinal flora fluid was obtained by adding a five-fold volume of PBS buffer and filtering the residue. Gifu anaerobic medium (GAM) broth was used in the incubation, as previously described [16]. BG was added immediately to the intestinal content suspension to yield a final concentration of 250 µM, and the incubation experiment was conducted in a 37 °C anaerobic incubator (5.02% CO_2_, 5.23% H_2_, 89.75% N_2_). The reactions were terminated by adding a five-fold volume of ice-cold acetonitrile at 0, 5, 15, and 30 min, and 1, 3, 6, 10, and 24 h. Incubations of BG in PBS were conducted in parallel with those of the negative control.

### 2.7. Isolation and Incubation of S9 Fraction in Liver and Intestines

Rat liver and intestine tissue were homogenized and centrifugated, according to our previous study [13], and the supernatant was collected as the S9 fraction and stored at −80 °C. The BCA protein assay kit was used to determine the protein concentration. B (0.3 μM, 3 μM, and 30 μM) was incubated with rat liver or intestinal S9 fractions at 37 °C for 60 min in the Tris-HCl-buffered system (pH 7.4), consisting of 2 mM of UDPGA, 10 mM of MgCl_2_, and 25 µg/mL of alamethicin. The reactions were terminated by adding 100 µL of ice-cold acetonitrile.

### 2.8. Uptake Studies Using Transporter-Expressing HEK293 Cells

BG uptake by OATP1B1, OATP1B3, and OATP2B1 was evaluated using transporter-transfected human embryonic kidney (HEK293) cells, which were constructed at Huiyuan Biotechnology Co., Ltd. (Shanghai, China). The cultivation of cells and the validation of transporter function were carried out using the method reported by our research group [17]. The cells were rinsed and equilibrated for 10 min. The uptake assays were initiated by adding HBSS containing 10 µM of BG, with or without rifampicin (positive inhibitor, 150 µM) or bile acids (1, 10, and 100 µM). Then, the cells were quickly washed with ice-cold HBSS and lysed by the addition of 300 µL of purified water after 10 min of incubation. The BCA protein assay kit was used to measure the protein concentration.

### 2.9. Determination of Baicalin and Baicalein

The LC-MS/MS method validated previously [18] was used, with moderate modification, to determine BG and B. A Shimadzu LCMS-8060 triple quadrupole tandem mass spectrometer (Kyoto, Japan) equipped with an LC-30AD high-performance liquid chromatographic system (Kyoto, Japan) was employed. Chromatographic separation was achieved via an AcclaimTM RSLC 120 C_18_ column (2.1 mm × 100 mm, 2.2 μm, Thermo Fisher Scientific, Waltham, MA, USA), with the temperature maintained at 40 °C. The mobile phase was 10 mM ammonium acetate solution in water containing 0.2% formic acid (A) and acetonitrile (B) at a flow rate of 0.6 mL/min. The gradient elution was initiated at 25% B, maintained for 0.5 min, increased to 75% B linearly in 2.8 min, maintained at 4.4 min, and decreased to 25% B to equilibrate the column for 0.6 min. MS detection was conducted via an electrospray ionization (ESI) source and quantitative analysis of multiple reaction monitoring (MRM) in positive ion mode. The instrument parameters were as follows: nebulizing gas flow, 3.0 L/min; interface voltage, 4 kV; interface temperature, 300 °C; heat block temperature, 400 °C; desolvation line (DL) temperature, 250 °C. The mass transitions for the quantification of MRM were *m*/*z* 447.2→271.1 for BG, *m*/*z* 455.0→165.1 for verapamil (internal standard), *m*/*z* 271.1→123.1 for B, and *m*/*z* 239.1→129.1 for 6-hydroxyflavonoid (internal standard). The standard curve ranges were 5.00 to 2000 ng/mL (BG) and 1.00 to 100 ng/mL (B) in rat plasma, bile, and urine; 25.0 to 50,000 ng/g (BG) and 5.00 to 500 ng/g (B) in homogenates of intestinal tissues, contents, and feces.

### 2.10. Data Analysis

The pharmacokinetic parameters were calculated using a non-compartmental model with Phoenix WinNonLin 8.1 (Pharsight, Mountain View, CA, USA) software. Statistical comparisons between the two groups of rats were evaluated using a Student’s two-tailed unpaired *t*-test, and a value of *p* < 0.05 was considered significant. All data were presented as the mean ± S.D. (*n* ≥ 3).

The uptake rate (UR) was normalized by the protein concentration of cell lysis. If the UR of BG in the transporter cells line compared to that in mock cells was greater than 2, and the uptake in the transporter-expressed cells was inhibited by more than 50% by rifampicin, BG was considered as the substrate for the examined uptake transporters. The UR (pmol/min/mg protein) was calculated as follows:

UR = C cell lysis / (T × C protein) × 1000

where C cell lysis (nM) is the substrate concentration, T (min) is the incubation time, and C protein (µg/mL) is the protein concentration in cell lysis.

## 3. Results

### 3.1. Biochemistry Parameters and Histopathologic Sections

**Figure 1:**
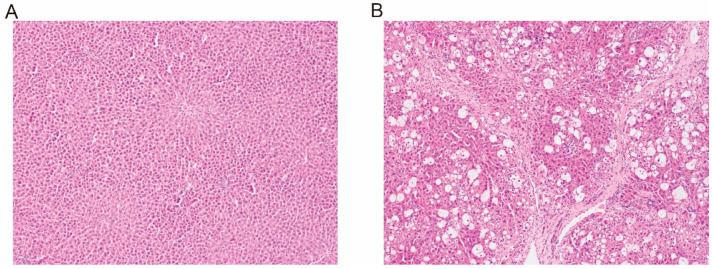
Liver histologic sections of control ((**A**) 100 ×) and CCl_4_-induced ((**B**) 100×) rats using hematoxylin–eosin staining.

After 8 weeks of treatment, the liver index and serum biochemical parameters (ALT, AST, AKP, and TBA) of the CCl_4_-induced rats were 1.32-, 8.28-, 2.23-, 2.82-, and 6.75-fold higher than those of the control rats, respectively (Table 1). The histopathology results with HE staining are shown in Figure 1. The liver structure of the control rats exhibited a relatively classical structure. In contrast, the livers of the CCl_4_-treated rats exhibited the signs of hepatocyte degeneration and necrosis, fibrous tissue infiltrated with inflammatory cells, and distortion of the central venules. These findings indicated that the CCl_4_-induced hepatic failure rat model was successfully established.

### 3.2. Pharmacokinetics of Baicalin and Baicalein in Rats

**Figure 2:**
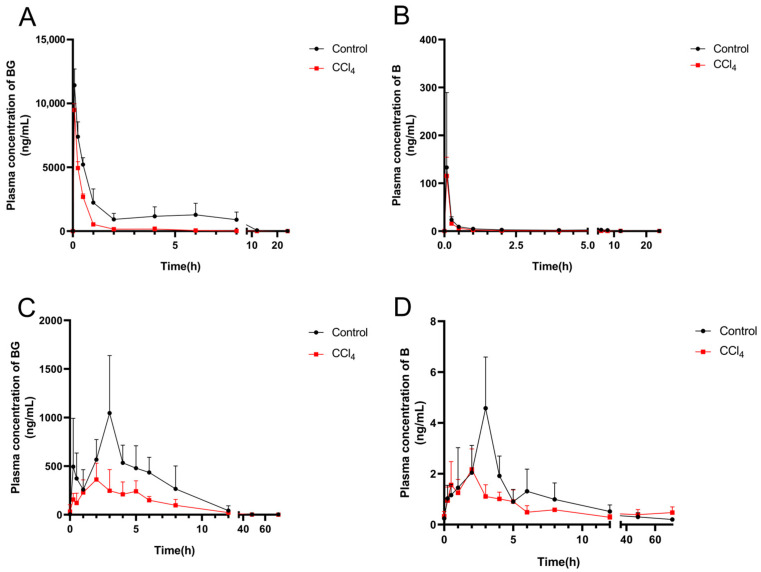
Mean plasma concentration–time curves of baicalin (**left**) and baicalein (**right**) after intravenous (**A**,**B**) or oral administration (**C**,**D**) of 10 mg/kg of baicalin to control and CCl_4_-induced rats. Each point is presented as mean ± S.D. (*n* = 5).

The plasma concentration–time profiles of BG and its aglycone B after an intravenous and oral administration of 10 mg/kg of BG are shown in Figure 2, and the pharmacokinetic parameters are listed in Table 2. After intravenous injection, BG was rapidly hydrolyzed into B, but its plasma exposure was less than 1% of BG. An evident absorption peak was observed in the plasma concentration–time curve of BG from the control rats, but not from CCl_4_-induced rats (Figure 2A). Compared to the control rats, the plasma exposure (AUC_0–t_) for BG and B in the CCl_4_-treated rats decreased by 73.6% and 46.3%, respectively. Following oral administration, the plasma peak concentration (C_max_) and AUC_0–t_ value of BG in the CCl_4_-induced rats decreased by 64.6% and 52.6%, respectively, compared with those of the control rats.

**Figure 3:**
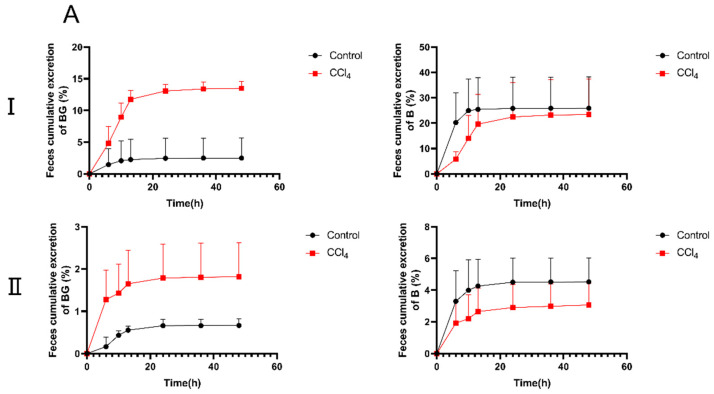

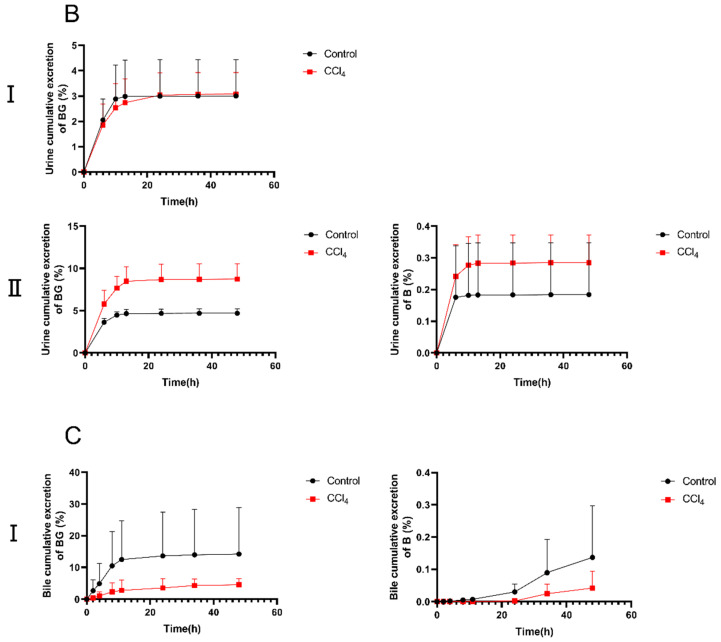
The percentage of cumulative excretion of baicalin (**left**) and baicalein (**right**) in feces (**A**), urine (**B**), and bile samples (**C**) after an oral (**I**) or intravenous (**II**) dose of 10 mg/kg of baicalin to control and CCl_4_-induced rats. Each point is presented as mean ± S.D. (*n* = 5).

To further elucidate the decreased absorption of BG in CCl_4_-induced rats, the excretion experiments were conducted. The cumulative excretion curves of BG and B in rat urine, feces, and bile are shown in Figure 3. Following an oral administration of 10 mg/kg of BG, the excretion of BG in the feces of CCl_4_-induced rats was significantly increased, from 2.50% to 13.5%, compared with the results for the control rats. However, the biliary excretion of BG in the bile duct-cannulated (BDC) rats from the CCl_4_ group decreased markedly, from 14.2% to 4.59%. Different from the results for oral administration, the urinary excretion of BG in CCl_4_-induced rats was significantly higher than that in control rats after intravenous administration of 10 mg/kg of BG (8.74% vs. 4.70%).

**Figure 4:**
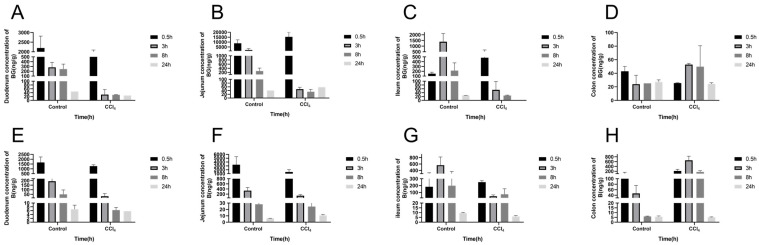
Concentration of baicalin (**A**–**D**) and baicalein (**E**–**H**) in the duodenum, jejunum, ileum, and colon after an oral administration of 10 mg/kg of baicalin to control and CCl_4_-induced rats. Each point is presented as mean ± S.D. (*n* = 3).

**Figure 5:**
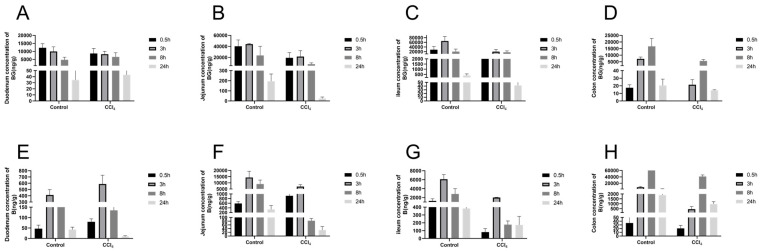
Concentration of baicalin (**A**–**D**) and baicalein (**E**–**H**) in the contents of the duodenum, jejunum, ileum, and colon after an oral administration of 10 mg/kg of baicalin to control and CCl_4_-induced rats. Each point is presented as mean ± S.D. (*n* = 3).

To evaluate the effect of liver injury on the mutual transformation of BG and aglycone B in the intestines, their concentrations in the intestinal tissues and their contents were analyzed (Figure 4 and Figure 5). Compared with the control rats, the exposures of BG and B were significantly decreased in the intestinal tissue and content of the CCl_4_-induced rats, especially in the jejunum, ileum, and their contents (Table S1).

### 3.3. Effect of Hepatic Impairment on the Hydrolysis Metabolism of Baicalin Mediated by Intestinal Microbiota

**Figure 6:**
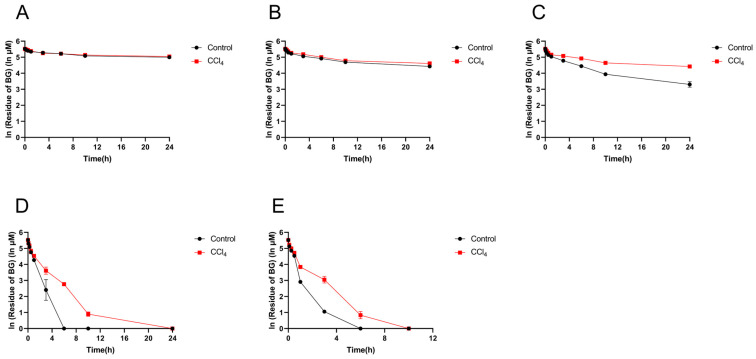
Residue of baicalin in the intestinal contents of the duodenum (**A**), jejunum (**B**), ileum (**C**), cecum (**D**), and colon (**E**) from control and CCl_4_-induced rats. Each point is presented as mean ± S.D. (*n* = 3).

To further verify the hydrolysis activity of the gut microbiota, the in vitro incubation experiment was carried out with 250 μM of BG as the substrate. As shown in Figure 6, the hydrolysis of BG occurred mainly in the contents of the cecum and the colon, followed by the ileum, and the hydrolysis of BG in the contents of the duodenum and jejunum was low. Compared with the control group, the hydrolysis metabolism rate of BG in the contents of the ileum, cecum, and colon from the CCl_4_-induced group decreased significantly. 

### 3.4. Effect of Hepatic Impairment on UGTs-Mediated Baicalein Metabolism to Baicalin

**Figure 7:**
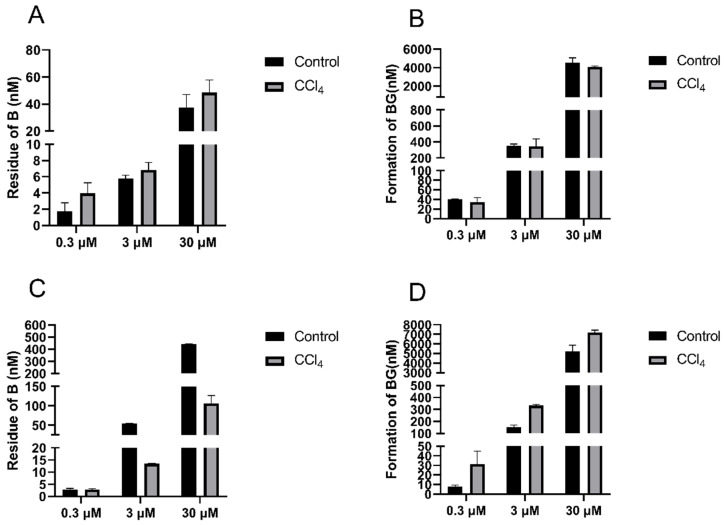
Residue of baicalein (**left**) and formation of baicalin (**right**) in liver S9 (**A**,**B**) and intestinal S9 (**C**,**D**) of control and CCl_4_-induced rats after incubation with 0.3, 3, and 30 μM of baicalein. Each point is presented as mean ± S.D. (*n* = 3).

To investigate the impact of hepatic impairment on the UGTs activities, B was incubated with liver S9 and intestinal S9 isolated from the control and CCl_4_-treated rats (Figure 7). The residue of B in the liver S9 of the CCl_4_-induced rats was 45.2%, 15.3%, and 22.9% higher than that in the control rats, and the formation of BG was 13.7%, 2.7%, and 10.3% lower, respectively, when 0.3, 3, and 30 μM of B were incubated, respectively. In contrast to the decrease in UGTs activity in the liver S9 of the CCl_4_-treated rats, there was a significant increase in the intestinal S9 fractions of the CCl_4_-treated rats, as evidenced by 24.1%, 20.3% and 28.6% increases in BG formation compared to the levels in the control rats.

### 3.5. Effect of Bile Acids on the Uptake of Baicalin

**Figure 8:**
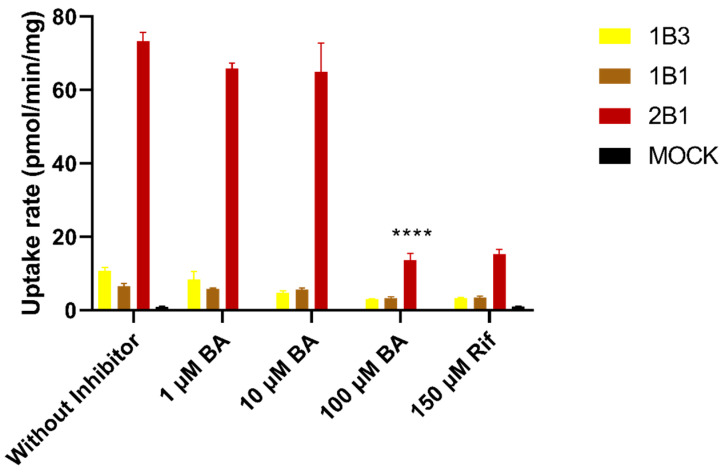
Uptake of 10 μM of baicalin and inhibitory effects of mixed bile acids in HEK293-OATP1B1, HEK293-OATP1B3, HEK293-OATP2B1, and HEK293-Mock. Data are expressed as mean ± S.D. (*n* = 3). Rif: rifampin, inhibitors of OATPs; BA: human mixed bile acids. **** *p* < 0.0001.

As shown in Figure 8, the uptake of 10 µM of BG into HEK293 cells transfected with OATP1B1, OATP1B3, and OATP2B1 were 7.3-, 12.0-, and 81.5-fold higher than that for empty-vector transfected cells, respectively. Rifampicin, a positive inhibitor of OATPs, significantly inhibited the uptake of BG, suggesting that BG is a substrate for OATPs, specifically, a sensitive substrate of OATP1B2, consistent with the literature report [5]. In the presence of 100 µM of human mixed bile acids, the activity of uptake transporter OATP2B1 was significantly inhibited.

## 4. Discussion

In the present study, the effects of hepatic impairment on the pharmacokinetics of BG were evaluated using a rat model of CCl_4_-induced hepatic failure. The findings demonstrated that, in contrast to the reported glucuronide metabolites of ezetimibe and morphine [17,19], the plasma exposure of BG in CCl_4_-induced rats was diminished by 73.6% and 52.6%, respectively, after an intravenous and oral administration of BG. It has been reported that BG undergoes extensive intestinal first-pass metabolism and enterohepatic circulation after intragastric administration [10,12,13]. Herein, the C_max_ of both BG and B was reduced by 64.6% and 54.4%, respectively, in CCl_4_-induced rats treated orally with BG. Conversely, the C_max_ of metabolically produced BG did not change when CCl_4_-treated rats were administered B by gavage (Table S2), indicating that the hydrolytic metabolism of BG in the intestine was diminished, leading to decreased absorption. This result was further supported by in vitro intestinal flora incubation assays, along with the distribution data from the intestinal tissues and contents. After the intravenous or intragastric administration of BG, there was no obvious second absorption peak on the concentration–time curve, suggesting that the enterohepatic circulation, which contributes significantly to the plasma exposure of BG, was considerably reduced in CCl_4_-induced rats.

BG has been identified as a substrate for the efflux transporters BCRP and MRP2, located on the bile canalicular membrane of the hepatocytes [4]. In the present study, the uptake transport results (Figure 8) also indicated that BG was a substrate of the uptake transporters (OATP1B1, 1B3, and 2B1) expressed on the basolateral membrane of the hepatocytes. As documented, hepatic impairment could lead to altered rat Mrp2 localization, impeding its capacity for drug and/or metabolite efflux into biliary excretion [20]. Additionally, hepatic impairment also caused the downregulation of rat Bcrp expression and a concomitant decrease in its transport activity [17]. However, for Oatps, CCl_4_-induced hepatic injury did not affect the uptake activity of rat Oatps, but the accumulation of bile acids due to liver injury could inhibit their uptake activities [17]. In this study, we observed the same phenomenon, i.e., that bile acids significantly inhibited the uptake activity of OATPs for BG, especially OATP2B1. Consequently, the reduced activities of the efflux and uptake transporters in the liver resulted in the obstruction of BG bile excretion. The recovery of BG in bile reduced from 14.3% to 4.63% in CCl_4_-treated BDC rats after an oral administration of 10 mg/kg of BG. Furthermore, although a small amount of BG might be excreted into the intestine via bile, the reduced β-glucuronidase activity of the gut microbiota prevented its hydrolysis into aglycone, as well as its subsequent reabsorption. This dual effects of the transporters and metabolism inevitably led to the decreased plasma exposure of BG in rats with liver injury.

Decreased hepatic transporter activity could result in a significant increase in plasma exposure of drugs or metabolites with predominant biliary excretion due to the impaired excretion, as evidenced by the glucuronide conjugates of EZE [17] and morphine [19]. In the present study, we found that after intravenous administration of 6.05 mg/kg B, a 39.0% increase in plasma exposure of BG (major component in the systemic circulation) was found in CCl_4_-induced rats, while the plasma exposure of B, a non-transporter substrate, was unaffected. This differed from the direct intravenous administration of BG, where BG plasma exposure decreased. As for this phenomenon, it could be explained that BG directly entered the systemic circulation after intravenous administration, while the activity of Oatps in the sinusoidal side was inhibited by bile acids due to liver injury, so that BG could not be taken into the liver. Conversely, following intravenous administration of B, B could enter the liver through passive diffusion and be metabolized to BG in the liver. Therefore, the plasma concentration–time curves (Figure 2A and Figure S1A) showed that the reduction in enterohepatic circulation in the BG group was greater than that in the B group. In addition, BG has been identified as a substrate for renal uptake transporter OATs [7]. It has been reported that OAT1 and OAT3 were compensatorily upregulated under cholestasis conditions [21,22]. When bile excretion was impeded due to liver injury, the kidneys compensated for the increase in renal excretion of BG. The present study also found that the urinary recovery of BG increased from 4.70% in the control group to 8.74% in the CCl_4_ group after intravenous administration of BG.

Zhang et al. constructed a rat model of acute liver injury with intrahepatic cholestasis using 17α-ethynyl-estradiol and evaluated the effects of such liver injury on the pharmacokinetics of BG. The results showed a 1.03-fold increase in plasma exposure following gavage administration of 200 mg/kg of BG [23], which was not in alignment with the results of our study. On the one hand, this difference might be due to the high dose administered, resulting in the saturation of absorption and enterohepatic circulation, with a limitation effect of hydrolysis by intestinal flora. On the other hand, it is not clear whether the decrease in the hydrolytic activity of the intestinal flora found here was due to CCl_4_ or to liver injury. Liu et al. reported that intestinal β-glucuronidase activity increased in diabetic rats, resulting in elevated oral exposure of BG [24]. In antibiotic-pretreated rats, plasma exposure of BG was reduced after oral administration [9]. Furthermore, the reduced absorption of BG in febrile rats might also be caused by the inhibition of intestinal β-glucuronidase activity [25]. Collectively, these findings implied that the pharmacokinetics of drugs whose absorption or enterohepatic circulation could be affected by the intestinal microbiota (e.g., BG) may be modified by diverse disease conditions.

Notably, there were certain limitations in extrapolating data from rats to humans. The findings of this study showed that liver injury in rats led to a decrease in the plasma exposure of baicalin. Both the literature and our experimental results showed that baicalin was also a substrate of multiple human transporters (OATPs, OATs, BCRP, and MRPs) [4,5,6,7,8]. Therefore, it can be predicted that patients with impaired liver function may also experience altered bile excretion of BG due to changes in transporter function or activity [26]. However, it remains inconclusive whether the reduced intestinal hydrolysis affected the enterohepatic circulation and oral absorption. Although the literature has shown that the human gut microbiota interacts with liver dysfunction [27] and is closely related to the hydrolysis of glucuronic acid conjugates [28], there are no reports indicating that hepatic dysfunction could affect the hydrolysis of glucuronide conjugates by gut microbiota.

## 5. Conclusions

In conclusion, hepatic impairment induced by CCl_4_ changed the disposition of BG in rats after both oral and intravenous administration via attenuating its enterohepatic circulation. On one hand, the activity of the gut microbiota responsible for the hydrolytic metabolism of BG was weakened, resulting in reduced BG absorption. On the other hand, hepatic impairment led to the accumulation of bile acids, which inhibits the activity of transporters responsible for the uptake of BG into the liver and its efflux into bile. The dual effects of the gut microbiota and the transporters depressed the enterohepatic circulation of BG, leading to a significant decrease in plasma exposure in hepatic-impaired rats. This study could provide a potential basis for clinical medication in patients with hepatic insufficiency and provide reference for the clinical development of drugs exhibiting enterohepatic circulation pharmacokinetic characteristics.

## Figures and Tables

**Table 1:** The liver index and biochemical parameters of control and CCl_4_-induced rats (*n* = 12).

Parameter	Control Rats	CCl_4_-Induced Rats
Liver index (g/kg)	33.1 ± 2.30	43.7 ± 6.0 ***
ALT (U/L)	23.2 ± 5.60	192 ± 21 ****
AST (U/L)	80.6 ± 14.2	180 ± 30 ****
AKP (U/L)	4598 ± 936	12,982 ± 887 ****
TBA (µmol/L)	39.1 ± 2.80	264 ± 61 ****

*** *p* < 0.001, **** *p* < 0.0001.

**Table 2:** Pharmacokinetic parameters of baicalin and baicalein after intravenous and oral administration of 10 mg/kg of baicalin to control (*n* = 5) and CCl_4_-induced rats (*n* = 5).

Route of Administration	Analyte	Pharmacokinetic Parameters	Control Rats	CCl_4_-Induced Rats
Intravenous	BG	C_5-min_ (ng/mL)	11,420 ± 1144	8484 ± 2047
		t_1/2_ (h)	2.42 ± 0.22	4.46 ± 2.17
		Ke (1/h)	0.288 ± 0.026	0.195 ± 0.098
		CL (mL/h/kg)	648 ± 167	2343 ± 197
		Vd (L/kg)	2.27 ± 0.65	15.4 ± 8.8
		AUC_0–t_ (h·ng/mL)	16,218 ± 3705	4278 ± 335
	B	C_max_ (ng/mL)	133 ± 140	94.8 ± 51.2
		t_max_ (h)	0.083	0.12 ± 0.07
		t_1/2_ (h)	6.16 ± 2.59	4.89 ± 1.35
		AUC_0–t_ (h·ng/mL)	55.7 ± 26.0	29.9 ± 2.76
Oral	BG	C_max_ (ng/mL)	1258 ± 390	445 ± 110
		t_max_ (h)	2.65 ± 1.26	2.60 ± 1.36
		t_1/2_ (h)	12.3 ± 9.03	8.00 ± 1.24
		AUC_0–t_ (h·ng/mL)	5055 ± 1411	2395 ± 888
	B	C_max_ (ng/mL)	5.04 ± 1.21	2.30 ± 0.66
		t_max_ (h)	2.60 ± 0.80	1.70 ± 0.60
		t_1/2_ (h)	39.9 ± 23.3	39.2 ± 11.2
		AUC_0–t_ (h·ng/mL)	32.6 ± 8.06	29.4 ± 13.2

AUC_0–t_, area under the concentration–time curve from 0 h to the last sampling time; CL, clearance; C_max_, maximum plasma concentration; Ke, elimination rate constant; t_max_, time to the C_max_; t_1/2_, apparent elimination half-life; Vd, volume of distribution.

## Data Availability

The authors confirm that the data supporting the findings of this study are available within the article.

## Supplementary Materials

The following supporting information can be downloaded at: https://www.mdpi.com/article/10.3390/pharmaceutics17070851/s1, Table S1: Mean AUCs following oral administration of 10 mg/kg baicalin; Table S2: Pharmacokinetic parameters of baicalin and baicalein after intravenous and oral administration of 6.05 mg/kg baicalein to control and CCl_4_-induced rats; Figure S1: Mean plasma concentration-time curves of baicalin (left) and baicalein (right) after intravenous (A,B) or oral administration (C) of 6.05 mg/kg baicalein to control and CCl_4_-induced rats.

## Abbreviations

The following abbreviations are used in this manuscript:

AKP	Alkaline phosphatase
ALT	Alanine aminotransferase
AST	Aspartate transaminase
AUC_0–t_	Area under the concentration–time curve
B	Baicalein
BCRP	Breast cancer resistance protein
BDC	Bile duct-cannulated
BG	Baicalin
CCl_4_	Carbon tetrachloride
C_max_	Plasma peak concentration
HE	Hematoxylin–eosin
HEK293	Human embryonic kidney 293 cells
LC-MS/MS	Liquid chromatography tandem mass spectrometry
MRPs	Multidrug resistance-associated proteins
OATs	Organic anion transporters
OATPs	Organic anion transporting polypeptides
S9	9000 g supernatant
UGTs	Uridine 5′-diphospho-glucuronosyltransferases

## References

[1] Hu Q., Zhang W., Wu Z., Tian X., Xiang J., Li L., Li Z., Peng X., Wei S., Ma X. (2021). Baicalin and the Liver-Gut System: Pharmacological Bases Explaining Its Therapeutic Effects. Pharmacol. Res..

[2] Su H.-X., Yao S., Zhao W.-F., Li M.-J., Liu J., Shang W.-J., Xie H., Ke C.-Q., Hu H.-C., Gao M.-N. (2020). Anti-SARS-CoV-2 Activities in Vitro of Shuanghuanglian Preparations and Bioactive Ingredients. Acta Pharmacol. Sin..

[3] Wen Y., Wang Y., Zhao C., Zhao B., Wang J. (2023). The Pharmacological Efficacy of Baicalin in Inflammatory Diseases. Int. J. Mol. Sci..

[4] Kalapos-Kovács B., Magda B., Jani M., Fekete Z., Szabó P.T., Antal I., Krajcsi P., Klebovich I. (2015). Multiple ABC Transporters Efflux Baicalin. Phytother. Res..

[5] Kalapos-Kovács B., Juhász V., Temesszentandrási-Ambrus C., Magda B., Szabó P.T., Antal I., Klebovich I., Krajcsi P. (2018). Baicalin Is a Substrate of OATP2B1 and OATP1B3. Phytother. Res..

[6] Zhang L., Li C., Lin G., Krajcsi P., Zuo Z. (2011). Hepatic Metabolism and Disposition of Baicalein via the Coupling of Conjugation Enzymes and Transporters—In Vitro and In Vivo Evidences. AAPS J..

[7] Xu F., Li Z., Zheng J., Gee Cheung F.S., Chan T., Zhu L., Zhuge H., Zhou F. (2013). The Inhibitory Effects of the Bioactive Components Isolated from *Scutellaria baicalensis* on the Cellular Uptake Mediated by the Essential Solute Carrier Transporters. J. Pharm. Sci..

[8] Tsai P.-L., Tsai T.-H. (2004). Pharmacokinetics of Baicalin in Rats and Its Interactions with Cyclosporin A, Quinidine and SKF-525A: A Microdialysis Study. Planta Medica.

[9] Kang M.J., Ko G.S., Oh D.G., Kim J.S., Noh K., Kang W., Yoon W.K., Kim H.C., Jeong H.G., Jeong T.C. (2014). Role of Metabolism by Intestinal Microbiota in Pharmacokinetics of Oral Baicalin. Arch. Pharmacal Res..

[10] Zhang L., Lin G., Kovács B., Jani M., Krajcsi P., Zuo Z. (2007). Mechanistic Study on the Intestinal Absorption and Disposition of Baicalein. Eur. J. Pharm. Sci..

[11] Xing J., Chen X., Sun Y., Luan Y., Zhong D. (2005). Interaction of Baicalin and Baicalein with Antibiotics in the Gastrointestinal Tract. J. Pharm. Pharmacol..

[12] Zhang L., Lin G., Zuo Z. (2007). Involvement of UDP-Glucuronosyltransferases in the Extensive Liver and Intestinal First-Pass Metabolism of Flavonoid Baicalein. Pharm. Res..

[13] Xing J., Chen X., Zhong D. (2005). Absorption and Enterohepatic Circulation of Baicalin in Rats. Life Sci..

[14] Kulik L., El-Serag H.B. (2019). Epidemiology and Management of Hepatocellular Carcinoma. Gastroenterology.

[15] Diep U., Chudow M., Sunjic K.M. (2017). Pharmacokinetic Changes in Liver Failure and Impact on Drug Therapy. AACN Adv. Crit. Care.

[16] Gotoh A., Nara M., Sugiyama Y., Sakanaka M., Yachi H., Kitakata A., Nakagawa A., Minami H., Okuda S., Katoh T. (2017). Use of Gifu Anaerobic Medium for Culturing 32 Dominant Species of Human Gut Microbes and Its Evaluation Based on Short-Chain Fatty Acids Fermentation Profiles. Biosci. Biotechnol. Biochem..

[17] Xie N., Wang H., Qin H., Guo Z., Xue H., Hu J., Chen X. (2022). Changes in Disposition of Ezetimibe and Its Active Metabolites Induced by Impaired Hepatic Function: The Influence of Enzyme and Transporter Activities. Pharmaceutics.

[18] Zhang S., Liu Y., Peng N., Chen X. (2022). Qualitative and Quantitative Determination of the Primary Active Components and Metabolites in Human Plasma after Oral Administration of Shuanghuanglian Liquid. J. Sep. Sci..

[19] Ferslew B.C., Johnston C.K., Tsakalozou E., Bridges A.S., Paine M.F., Jia W., Stewart P.W., Barritt A.S., Brouwer K.L.R. (2015). Altered Morphine Glucuronide and Bile Acid Disposition in Patients with Nonalcoholic Steatohepatitis. Clin. Pharmacol. Ther..

[20] Kojima H., Sakurai S., Yoshiji H., Uemura M., Yoshikawa M., Fukui H. (2008). The Role of Radixin in Altered Localization of Canalicular Conjugate Export Pump Mrp2 in Cholestatic Rat Liver. Hepatol. Res..

[21] Brandoni A., Villar S.R., Picena J.C., Anzai N., Endou H., Torres A.M. (2006). Expression of Rat Renal Cortical OAT1 and OAT3 in Response to Acute Biliary Obstruction. Hepatology.

[22] VanWert A.L., Gionfriddo M.R., Sweet D.H. (2010). Organic Anion Transporters: Discovery, Pharmacology, Regulation and Roles in Pathophysiology. Biopharm. Drug Dispos..

[23] Zhang C.-L., Xu Y.-J., Xiang D., Yang J.-Y., Lei K., Liu D. (2018). Pharmacokinetic Characteristics of Baicalin in Rats with 17α-Ethynyl-Estradiol-Induced Intrahepatic Cholestasis. Curr. Med. Sci..

[24] Liu L., Deng Y.-X., Liang Y., Pang X.-Y., Liu X.-D., Liu Y.-W., Yang J.-S., Xie L., Wang G.-J. (2010). Increased Oral AUC of Baicalin in Streptozotocin-Induced Diabetic Rats Due to the Increased Activity of Intestinal Beta-Glucuronidase. Planta Medica.

[25] Huo X.-K., Wang B., Zheng L., Cong H.-J., Xiang T., Wang S.-M., Sun C.-P., Wang C., Zhang L., Deng S. (2017). Comparative Pharmacokinetic Study of Baicalin and Its Metabolites after Oral Administration of Baicalin and Chaiqin Qingning Capsule in Normal and Febrile Rats. J. Chromatogr. B.

[26] Lake A.D., Novak P., Fisher C.D., Jackson J.P., Hardwick R.N., Billheimer D.D., Klimecki W.T., Cherrington N.J. (2011). Analysis of Global and Absorption, Distribution, Metabolism, and Elimination Gene Expression in the Progressive Stages of Human Nonalcoholic Fatty Liver Disease. Drug Metab. Dispos..

[27] Liu S., Yang X. (2023). Intestinal Flora Plays a Role in the Progression of Hepatitis-Cirrhosis-Liver Cancer. Front. Cell. Infect. Microbiol..

[28] Li P., Zhang R., Zhou J., Guo P., Liu Y., Shi S. (2024). Vancomycin Relieves Tacrolimus-Induced Hyperglycemia by Eliminating Gut Bacterial Beta-Glucuronidase Enzyme Activity. Gut Microbes.

