# Supplementary material for: Effect of Hepatic Impairment on the Pharmacokinetics of Baicalin in Rats: Critical Roles of Gut Microbiota and Hepatic Transporters
Source: Pharmaceutics. 2025 Jun 29;17(7):851. doi: 10.3390/pharmaceutics17070851 (PMC12298447; doi:10.3390/pharmaceutics17070851)

Supplementary

Table S1. Mean AUCs following oral administration of 10 mg/kg baicalin

Group	Intestine	Intestinal segments		Intestinal contents	
		AUC_{0-24h} of BG (ng/g*h)	AUC_{0-24h} of B (ng/g*h)	AUC_{0-24h} of BG (ng/g*h)	AUC_{0-24h} of B (ng/g*h)
Control	Duodenum	3968	1849	80694	3074
	Jejunum	12629	2931	387065	118516
	Ileum	6038	3448	364452	39104
	Colon	125	271	180260	571294
	Total	22760	8498	1012471	731988
CCl_4	Duodenum	978	797	71525	3119
	Jejunum	7984	1401	127520	22395
	Ileum	555	886	173333	8312
	Colon	582	3478	44138	319767
	Total	10099	6562	416517	353593

Table S2. Pharmacokinetic parameters of baicalin and baicalein after intravenous and oral administration of 6.05 mg/kg baicalein to control and CCl_4-induced rats

Route of Administration	Compound	Pharmacokinetic Parameters	Control rats	CCl_4-induced rats
i.v.	BG	C_{max}(ng/mL)	3832±543	4720±956
		t_{max}(h)	0.12±0.06	0.15±0.08
		$t_{1/2}$ (h)	3.18±0.23	3.95±1.16
		AUC_{0-t} (h*ng/mL)	2697±501	3751±1022
	B	$C_{5\ min}$(ng/mL)	5020±752	5380±925
		$t_{1/2}$ (h)	8.95±2.06	7.77±5.67
		AUC_{0-t} (h*ng/mL)	929±318	875±126
p.o.	BG	C_{max}(ng/mL)	2058±911	1989±1007
		t_{max}(h)	0.30±0.10	0.35±0.12
		$t_{1/2}$ (h)	2.52±0.92	3.68±1.10
		AUC_{0-t} (h*ng/mL)	6996±2474	4072±1365

Each dot was presented as mean ± S.D. of value from control rats (n = 5) or CCl_4- induced rats (n = 5).

Figure S1. Mean plasma concentration-time curves of baicalin (left) and baicalein (right) after intravenous (A, B) or oral administration (C) of 6.05 mg/kg baicalein to control and CCl_4-induced rats. Each point was presented as mean ± S.D. (n=5).

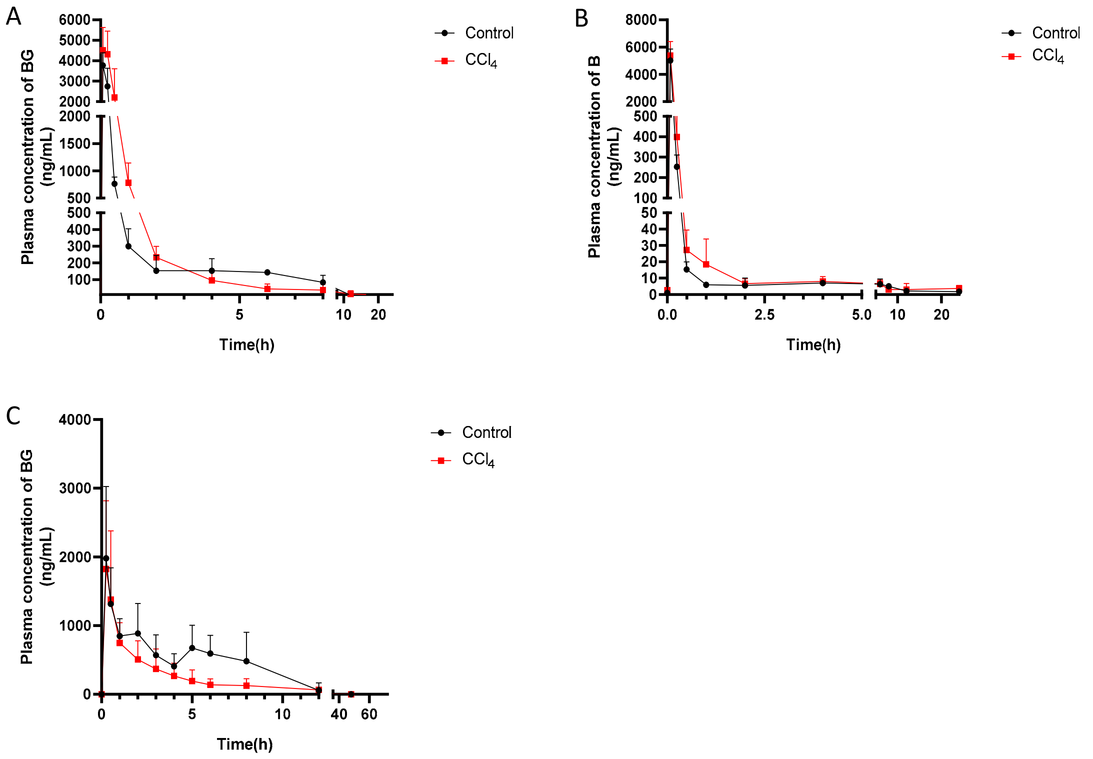

Supplement: Supplementary file 1 [file pharmaceutics-17-00851-s001.zip › pharmaceutics-3681925-supplementary.pdf]
